# Bullous pemphigoid: The role of type 2 inflammation in its pathogenesis and the prospect of targeted therapy

**DOI:** 10.3389/fimmu.2023.1115083

**Published:** 2023-02-16

**Authors:** Luyao Zhang, Zihua Chen, Lanting Wang, Xiaoqun Luo

**Affiliations:** Department of Allergy and Immunology, Huashan Hospital, Fudan University, Shanghai, China

**Keywords:** type 2 inflammation, bullous pemphigoid, immunoglobulin E, eosinophils, targeted therapy

## Abstract

Bullous pemphigoid (BP) is an autoimmune disease that mainly occurs in the elderly, severely affecting their health and life quality. Traditional therapy for BP is mainly based on the systemic use of corticosteroids, but long-term use of corticosteroids results in a series of side effects. Type 2 inflammation is an immune response largely mediated by group 2 innate lymphoid cells, type 2 T helper cells, eosinophils, and inflammatory cytokines, such as interleukin (IL)-4, IL-5 and IL-13. Among patients with BP, the levels of immunoglobulin E and eosinophils are significantly increased in the peripheral blood and skin lesions, suggesting that the pathogenesis is tightly related to type 2 inflammation. To date, various targeted drugs have been developed to treat type 2 inflammatory diseases. In this review, we summarize the general process of type 2 inflammation, its role in the pathogenesis of BP and potential therapeutic targets and medications related to type 2 inflammation. The content of this review may contribute to the development of more effective drugs with fewer side effects for the treatment of BP.

## 1 Introduction

Bullous pemphigoid (BP) is one of the most frequent autoimmune bullous diseases that mainly occurs in the elderly and display no gender predilection. The cumulative incidence of BP is 8.2/million individuals, which is higher in Europe than in Asia (1). Although the incidence is generally low, the risk of developing BP increases with age. Studies have shown that people over 90 years of age have a 300-fold increased risk compared to people under 60 years of age (2). Moreover, compared to the general population of the same age, patients with BP have a 3.6 times increased risk of death (3). Although it is an autoimmune disease, it can be induced by multiple stimuli, including gliptin, COVID-19 vaccines, and programmed cell death-1/programmed cell death ligand-1 inhibitors (4–6), suggesting possible different biological underpinnings in these patients.

BP is predominantly evoked by autoantibodies against two types of hemidesmosomal proteins, BP180 (XVII collagen) and BP230, which are located in the basement membrane zone (BMZ) and responsible for the dermo-epidermal junction (7). During the formation of subepidermal blisters with negative Nikolsky sign, these autoantibodies and immune cells act together to destroy hemidesmosomes in keratinocytes (8). Histopathological examination frequently reveals separation of the dermis and epidermis, and inflammatory cell infiltration, mainly composed of lymphocytes and eosinophils (9).

BP cause a vast array of burdens to patients. Patients with BP usually develop pruritic, tense blisters or bullae locally or widespread on normal skin or erythematous background on the trunk and limbs (10). Some patients present with a non-bullous prodromic phase characterized by eczematous, excoriated, urticaria-like, or nodular lesions that varying in duration (11). Meanwhile, patients often suffer from comorbid health conditions, including neurological disorders, malignancies, and cardiovascular diseases. Specifically, it has been proven that multiple sclerosis, diabetes, hypertension, basal cell carcinoma of the skin, dementia, Parkinson’s disease, epilepsy, stroke, pneumonia, and pulmonary embolism have an increased prevalence among patients with BP (11–15). In addition to physical discomfort, BP could also contribute to decreased quality of life and increased psychological burden, such as anxiety and depression, because of the skin lesions, functional problems, pruritus, and disease chronicity (16, 17).

In terms of therapy, topical or systematic glucocorticosteroids are the primary treatment for BP, which can be supplemented by immunosuppressors such as methotrexate, azathioprine, and mycophenolate mofetil (2). However, long-term systemic use of corticosteroids may cause a variety of side effects, including hypertension, bone fracture, cataract, gastrointestinal discomfort and metabolic conditions, such as weight gain and hyperglycemia (18). Another key fact is that some patients with BP are resistant to traditional treatment. Currently, plasmapheresis, intravenous immunoglobulin, immunoadsorption, and rituximab can be administered to refractory patients (11). It has been demonstrated that more than 90% of patients with moderate-to-severe BP can get complete remission relatively safely using a combination therapy with rituximab and corticosteroids (19). However, some patients with BP are not sensitive to these therapies; thus, there is an urgent need for drugs with fewer side effects and superior efficacy.

Recently, an increasing number of studies have found that the pathogenesis of BP is closely related to type 2 inflammation, and the use of dupilumab, a monoclonal antibody against the type 2 inflammatory factors interleukin 4 (IL-4) and IL-13, seems to have a certain curative effect in patients with BP. Moreover, the European Academy of Dermatology and Venereology considers that dupilumab and omalizumab are optional treatments for refractory BP (20). These results provide a new direction for further exploration of the pathogenesis of BP and the search for more effective treatments. Here, we review the general process of type 2 inflammation, its role in the BP pathogenesis, and potential therapeutic targets and medications related to type 2 inflammation. Our aim is to provide new ideas and research directions for the development of more effective drugs with fewer side effects for the treatment of BP.

## 2 The general process of type 2 inflammation

Type 2 inflammation is an immune response which exerts an important role in host defense against parasites and is predominantly mediated by group 2 innate lymphoid cells (ILC2s), type 2 T helper (Th2) cells, eosinophils, and relevant cytokines, such as IL-4, IL-5, and IL-13 (21). A large number of stimuli can trigger type 2 inflammation, including helminths, various allergies, certain viral or bacterial infections, and endogenous molecules (22). The process involves both innate and adaptive immune responses.

After exposure of the epithelium to these stimuli, local tissue homeostasis is disrupted, and epithelial cells release IL-25, IL-33, and thymic stromal lymphopoietin (TSLP), which are termed alarmins. In general, epithelial tissues in different parts of the body release various alarmins. For example, in the lung, type 2 alveolar cells are a primary source of TSLP and IL-33 in the lung (23), whereas in the small intestine and skin, tuft cells and keratinocytes are a major source of IL-25, respectively (24, 25). In addition, other non-epithelial cells can also produce alarmins, with airway smooth muscle cells secreting TSLP (26) and fibroblasts producing IL-33 (27). Under the effect of these alarmins, tissue-resident ILC2s are activated and increase the production of cytokines of IL-4, IL-5, IL-9 and IL-13 (28), termed type 2 cytokines. ILC2 can also be mediated by other molecules released after the attack of exogenous stimuli, such as TL1A from TNF-family (29, 30), prostaglandin D2 and cysteinyl leukotriene (31, 32).

Meanwhile, in the presence of stimuli and cytokines produced by ILC2s, dendritic cells (DC) take up and transport parts of the antigens to local draining lymph nodes. Subsequently, the processed antigens induce naïve CD4+ T cells to activate the latter in the lymph nodes. The latter, with high GATA-binding protein 3 expression, can proliferate and differentiate into Th2 cells and subsequently produce type 2 cytokines with the help of DCs and the aforementioned type 2 cytokines, also including IL-33 and TSLP (21, 33, 34). After activation of ILC2s, this process generates as part of adaptive immunity. Furthermore, ILC2s have been shown to modulate the differentiation of naïve T cells into Th2 cells and the degree of Th2 response to a large extent. Once the stimulus is gone, ILC2s will be negatively regulated and decrease the support to Th2 cells, ultimately reducing the inflammation (35–37).

Generally, eosinophils, which develop in the bone marrow and circulate in the peripheral blood, play a vital role in type 2 inflammation. IL-5 is a crucial cytokine for the maturation, survival as well as recruitment of eosinophils and can prevent apoptosis (38). In type 2 inflammation, eosinophils are activated and recruited by IL-5, together with IL-33, TSLP, and eotaxins released by inflamed tissues and other chemoattractants, such as C3a and C5a (39–41). After activation, eosinophils can function as antigen-presenting cells for viral antigens (42) and upregulate associated molecules, including major histocompatibility complex (MHC) II, CD86, and CD40, ultimately leading to T cells activation and differentiation in draining lymph nodes (43). In the meantime, mature eosinophils can secrete IL-4, IL-25, and indoleamine 2,3-dioxygenase (44), which may cause selective differentiation of naïve T cells into Th2 cells instead of Th1 cells. Moreover, eosinophil-derived neurotoxin (EDN) and eosinophil peroxidase (EPO) released by eosinophils could strongly affect the maturation of DCs that migrate to lymph nodes, which can indirectly promote Th2 cell functions (45). Eosinophil-derived IL-25 can also promote the proliferation of and cytokine production by Th2 memory cells after antigen triggering (46). Besides their role as promoter for Th2 response, eosinophils can also act as an effector in type 2 inflammation. After recruitment into peripheral tissues, eosinophils cause tissue damage by releasing eosinophilic cationic protein (ECP), which can prompt cell cytotoxicity; EPO, which can generate oxidative stress; and major basic protein (MBP), which can destroy the lipid bilayer and increase cell permeability to damage the epithelium (43). Additionally, eosinophils are able to modulate classical and alternative pathways of complement activation as well as act through antibody-dependent cellular cytotoxicity against stimuli *via* their Fc receptors (43).

**Figure 1:**
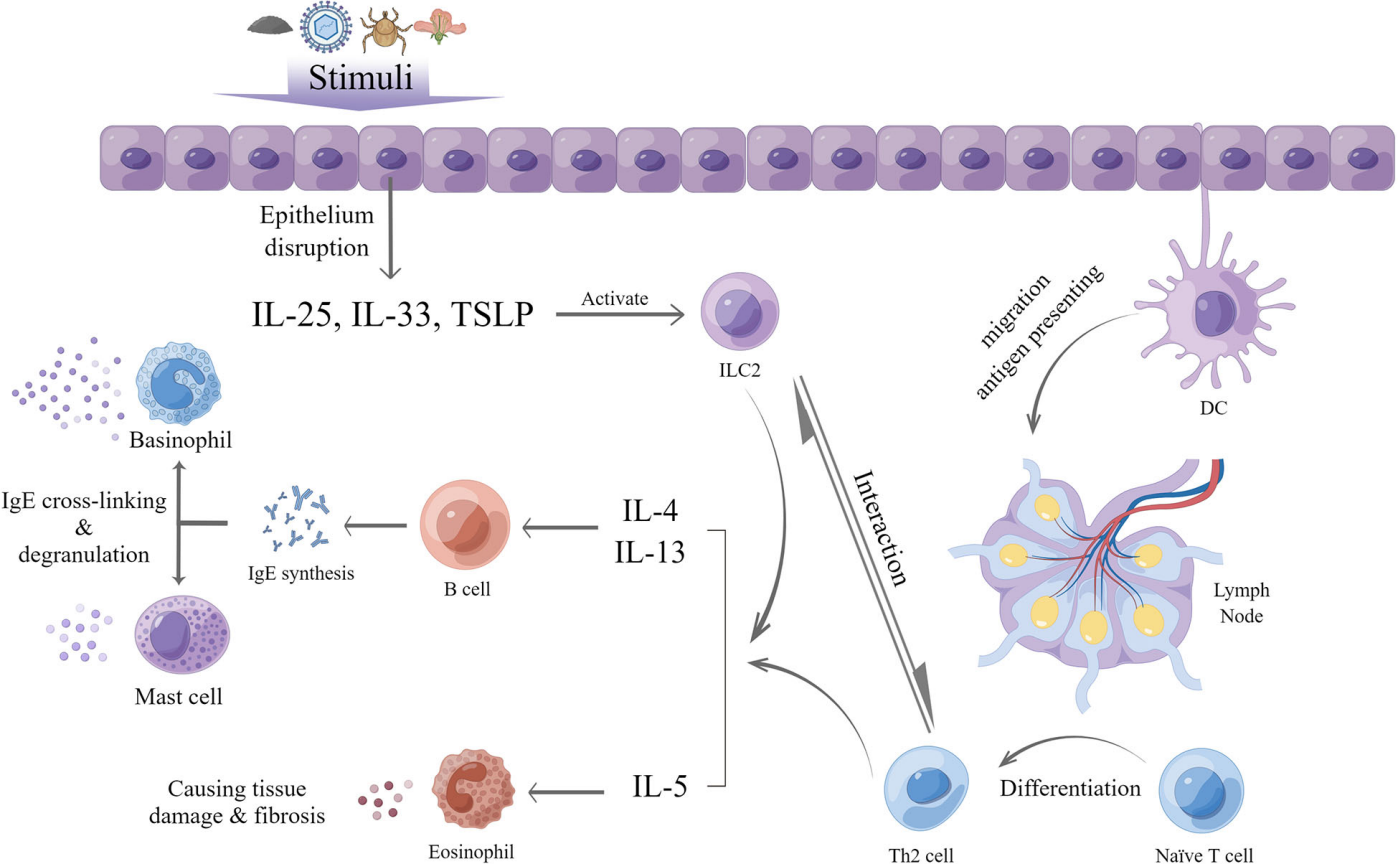
General process of type 2 inflammation (by Figdraw).

In the last phase of type 2 inflammation, the presence of stimuli and cytokines produced by both ILC2s and Th2 cells, especially IL-4 and IL-13, can facilitate the humoral immune response, activate B cells and cause rearrangement of the immunoglobulin heavy chain locus thus leading to immunoglobulin E (IgE) synthesis and differentiation into IgE-producing plasma cells (47, 48). IgE can bind to its high affinity receptor, FcϵRI, on basophils and mast cells to sensitize the body. When the body is exposed to the corresponding allergen again, the antigen recognition fragment of IgE binding to the cell surface binds to the allergen, resulting in cross-linking with the IgE-FcϵRI complex and degranulation of mast cells and basophils. The degranulation products, including histamine, proteases, prostaglandins and other cytokines (49, 50), lead to inflammatory reactions that manifest as erythema, itching, and edema on the skin; sneezing; cough; increased mucous secretions and bronchospasm in the respiratory tract; nausea, vomiting, and diarrhea in the digestive tract; and hypotension (49) (**Figure 1**)

## 3 Type 2 inflammation in BP and potential therapeutic targets

As previously mentioned, BP is predominantly evoked by autoantibodies against BP180 and BP230. In these patients, BP180 and BP230 are recognized, ingested, processed by antigen presenting cells, and subsequently expressed on the cell surface combined with MHC II. After recognition of the antigen, naïve T cells are activated, differentiate to autoreactive Th2 cells and secrete specific cytokines, which in turn stimulate the differentiation and class-switch recombination of B cells. Plasmocytes produce autoantibodies IgG and IgE, leading to the deposition of autoantibodies in both the peripheral blood and the local basement membrane zone. Therefore, Th2 pathways are considered the primary triggers for antibody production in BP (51). It seems that anti-BP180 antibodies are more common than anti-BP230 antibodies in BP. One study showed that anti-BP180 IgG can be found in 95% of patients with BP, while anti-BP230 was found in 70% of patients in the same group (52). Autoantibody IgG can trigger complement activation, which can be proven by the deposition of complement C3 and C4 found in immunofluorescence examination, multiple immune cells recruitment, and proteases release, which can cause an inflammatory cascade reaction. Furthermore, it can directly target the corresponding antigens, causing hemidesmosome destruction and leading to the loss of adhesion to the BMZ and formation of subepidermal blisters (8). Among these autoantibodies, anti-BP180 antibodies are mainly targeted to the immunodominant region termed NC16A in the extracellular regions (53). These anti-BP180 NC16A IgG are positively correlated with the Autoimmune Bullous Skin Disorder Intensity Score and Bullous Pemphigoid Disease Area Index (BPDAI) in its subcomponents of erosion/blister, urticaria/erythema and pruritus scores, while the level of anti-BP230 IgG is not correlated with these scores, suggesting that anti-BP180 NC16A IgG can be a useful indicator of BP activity (54, 55). The above mechanism can partially explain the formation of blisters but fail to explain the manifestation of itching, erythema, and eosinophilia in BP. Elevated serum IgE levels are found in 70–85% patients with BP (56), and deposition of IgE can be seen in immunofluorescence examination, which is related to degranulation of mast cells and basophils, eosinophil recruitment, and manifestations of itch and blister formation (57). Therefore, abnormal T cell immune responses and autoantibodies together lead to the occurrence of BP. The pathogenesis of BP depends on a variety of immune cells, including Th2 cells and eosinophils; autoimmune antibodies, including IgE; and a variety of cytokines, such as IL-4, IL-5, and IL-13, which are all relevant to type 2 inflammation.

### 3.1 Role of type 2 cytokines in BP

A variety of studies have revealed that the levels of IL-4, IL-5, IL-6, IL-10, and IL-13, which are predominantly produced by ILC2 and Th2 cells, are elevated in the serum, blister fluid, and skin biopsies of patients with BP (58–60). Among them, IL-4 is considered to have the closest association with BP (61) because it is particularly essential for Th2 cell differentiation, class-switch recombination of B cells, and production of IgE while simultaneously suppressing Th1 and Th17 differentiation (62). Subsequently, Th2 cells continuously produce more IL-4, IL-5, and IL-13 to further enhance this process. IL-5 is a critical cytokine for the maturation, survival, and functional activity of eosinophils, and its level parallels the severity of BP (60). IL-13 is another crucial cytokine in BP and has some common features with IL-4. They share a common receptor subunit of IL-4 receptor α (IL-4Rα) and a common intracellular signaling pathway (63); thus, they can synergistically promote B cell differentiation and IgE production. IL-13 levels also have a positive correlation with the itch severity of BP (64). In addition, BP patients tend to show a lower frequency of the C allele in IL-13 gene variation (rs1800925) and the G-allele in IL-4R rs1805010 than healthy individuals, suggesting their protective effects to BP (65). However, A-allele in IL-13 rs20541 can function as a promoting factor to the susceptibility of BP (65).

Owing to their significance in BP, several targeted medications have emerged. Dupilumab is an IL-4Rα antagonist that can block the common IL-4Rα subunit to inhibit IL-4 and IL-13 signaling (66). It was approved for the treatment of atopic dermatitis (AD) in 2017 and is currently being studied for many other type 2 inflammatory diseases, including BP. Recently, some studies have shown that dupilumab exerts favorable therapeutic effects in patients with BP (67–74). It can result in rapid improvement of skin lesions and pruritus, leading to complete remission or a satisfactory treatment response in 92.3% of patients, including those who do not respond to traditional treatments (67). Additionally, a phase 2/3 randomized double-blind placebo-controlled trial for its use in BP is currently underway (NCT04206553).

Mepolizumab and reslizumab are humanized monoclonal antibodies targeting IL-5. Mepolizumab has been used to treat eosinophilic granulomatosis with polyangiitis, eosinophilic asthma, and chronic rhinosinusitis with nasal polyps in clinical trials, and has shown efficacy and safety (75–79). However, in a phase 2 pilot study of mepolizumab for BP, there was no significant difference in the cumulative rates of patients who achieved and maintained disease control between the mepolizumab and placebo groups, indicating that the primary endpoint was not met (80). Similar to mepolizumab, reslizumab has been approved as an add-on treatment for adults with severe eosinophilic asthma (81–83). A study of reslizumab use in BP reported that reslizumab can rapidly improve bullous skin lesion (84). Further research is needed to confirm the effects of anti-IL-5 treatment on BP.

Benralizumab is a monoclonal antibody against the IL-5-receptor licensed for treating severe eosinophilic asthma (85). It is also used in the treatment of eosinophilic granulomatosis with polyangiitis, and for the prevention of chronic obstructive pulmonary disease exacerbations (86, 87). Tralokinumab and lebrikizumab are monoclonal antibodies targeting IL-13. Tralokinumab can bind to IL-13, inhibiting its interaction with certain receptors and thereby neutralizing its biological activity (88). It has been approved for moderate to severe AD by the European Commission (89). Lebrikizumab is currently used for the treatment of asthma and AD (90–94). It has a satisfactory curative effect on asthma, especially in terms of the pulmonary function and exacerbation rates (93). Although it has not been studied whether benralizumab, tralokinumab, and lebrikizumab are effective for BP, based on the significance of IL-5 and IL-13 in its pathogenesis, they may potentially be used for the treatment of BP in the future (**Table 1**).

**Table 1:** Therapeutic targets and medication related to type 2 inflammation in BP.

Medication	Therapeutic target	Research status	
		In BP	In other type 2 inflammatory diseases
Dupilumab	IL-4R	Phase 2/3 clinical trial	Approved for AD
Mepolizumab	IL-5	Phase 2 clinical trial	Using in therapy of eosinophilic granulomatosis with polyangiitis, eosinophilic asthma and chronic rhinosinusitis with nasal polyps
Reslizumab	IL-5	A case report showed its therapeutic effects	Approved for eosinophilic asthma
Benralizumab	IL-5R	/	Using in therapy of eosinophilic granulomatosis with polyangiitis and in the prevention of chronic obstructive pulmonary disease exacerbations
Tralokinumab	IL-13	/	Approved for moderate-to-severe AD
Lebrikizumab	IL-13	/	Using in therapy of asthma and AD
Bertilimumab	Eotaxin-1	Phase 2 clinical trial	/
AKST4290	CCR3	Phase 2 clinical trial	/
Nemolizumab	IL-31RA	/	Using in therapy of AD and prurigo nodularis
Omalizumab	IgE	Phase 3 clinical trial	Approved for severe persistent asthma and chronic idiopathic or spontaneous urticaria
Ligelizumab	IgE	Phase 2 clinical trial	Using in therapy of chronic spontaneous urticaria
Tezepelumab	TSLP	/	Using in therapy of asthma

### 3.2 Eosinophils and related molecules in BP

Eosinophil infiltration in skin lesions is a prominent feature of BP. They are recruited by many chemoattractants, including IL-5, eotaxin, and galectin-9, which are detected in the blister fluid (95). Studies have found that the level of eosinophils is elevated in the peripheral blood of over 50% of untreated patients with BP, which has been proven to be positively correlated with both disease and itch severity. Along with increased expression of CD69, eosinophils in both the blood and blister fluid are strongly activated (96–100). BP patients with eosinophilia tend to be older and have higher palmoplantar involvement than others (98). In patients with total IgE levels > 400 IU/mL, the level of eosinophils in peripheral blood is strongly related to the level of anti-BP180 IgE (101). The concentrations of ECP, EDN, and MBP, released by activated eosinophils, are higher in the serum and blister fluid of patients with BP than in the healthy controls (97). ECP and EDN can disrupt keratinocyte cell-matrix detachment, contributing to blister formation through ribonuclease activity (97, 102). ECP also affects the proliferation of T and B cells, promotes the degranulation of mast cells, and modulates the complement pathway (103). Eosinophils are considered the major source of IL-31 in BP, and the latter is a generally acknowledged pruritogen as well as a promoting factor for blister formation (102, 104, 105). The level of IL-31 in the serum is significantly associated with the level of anti-BP180 IgE (97). In addition, activated eosinophils can release metalloproteinase-9, which can lead to BP180 cleavage (106). In an *in vitro* study, eosinophils activated by IL-5 were shown to degranulate and directly cause blister formation in the presence of BP autoantibodies after adhesion to keratinocytes and FcγR activation (107). Furthermore, eosinophils have been proven to be correlated with blood coagulation in BP, since it has been proven that the level of ECP in blister fluid is elevated and paralleled to markers of coagulation activation (108). Eosinophils are considered a source of tissue factor and the latter can initiate blood coagulation cascade, manifested as elevated prothrombin fragment F1 + 2 and D-dimer levels in both the plasma and blister fluid, which may contribute to inflammation, tissue damage, blister formation, and thrombotic risk (109–111). Although in some studies, a lower eosinophil count is considered a potential risk factor for mucosal involvement (112, 113), elevated eosinophil levels are generally considered a marker of disease severity in BP, and targeting eosinophils may be a promising treatment for BP. Bertilimumab is a humanized monoclonal antibody targeting eotaxin-1. In a phase 2 clinical trial of BP, the disease severity decreased by 81% after 13 weeks of use of bertilimumab (114). AKST4290 is an antagonist of CCR3, the major receptor of eotaxin on eosinophils. In a phase 2 study of AKST4290, patients with BP were administered 400 mg AKST4290 twice together with mometasone furoate until the disease was under control (115). Nemolizumab, which is a monoclonal antibody against IL-31 receptor A, is licensed to treat AD and prurigo nodularis and can observably reduce pruritus (116). Based on the role of IL-31 in itching, nemolizumab may potentially be used in BP as an additional medication to control pruritus in the future (**Table 1**).

### 3.3 IgE in BP

As mentioned previously, IgE is critical for the occurrence and development of type 2 inflammation. In patients with BP, elevated IgE levels in the serum and deposition of IgE in the BMZ were first described in 1974 (117). Since then, an increasing amount of evidence has suggested that IgE is essential for the pathogenesis of BP. Later, researchers found that some IgE autoantibodies target the NC16A region of BP180, which is the same as anti-BP180 IgG (118), although the incidence of anti-BP180 IgE varies widely among patients. Other studies have detected BP230-specific IgE in BP (119, 120). The clinical features of BP are associated with IgE levels. The deposition of IgE in the BMZ is parallel to BPDAI scores and disease course (121). Patients with pathological findings of linear deposition of IgE in the BMZ tend to have higher levels of anti-BP180 IgE in the serum than patients without IgE deposition (121). Many studies have reported that the levels of total IgE in the serum are positively related to disease severity in patients with elevated IgE levels (122–124). Some studies have found that the level of anti-BP180 IgE in the serum is positively associated with BPDAI scores (124). It has also been reported that the level of anti-BP230 IgE is associated with disease activity or local eosinophil infiltration (125, 126). Moreover, anti-BP230 IgE is more common in patients resistant to topical corticosteroids, suggesting that it can be used as a marker for systemic corticosteroid therapy (127). In addition, *in vitro* experiments have shown that anti-BP180 IgE can cause a decline in keratinocyte adhesion and hemidesmosomal density (128), suggesting its function in blister formation. IgE receptors are increased as well in BP. It has been found that the expression of CD23, a receptor of IgE, is increased on peripheral B cells and correlates with IgE levels as well as disease severity (129, 130). Meanwhile, galectin-3, a soluble receptor for IgE, has lower expression around blisters in BP, which may contribute to the extension of blisters by disassembling the cell-extracellular matrix (131).

Recently, there have been several therapies aimed at increasing IgE levels. Omalizumab, a monoclonal anti-IgE antibody, has been approved for treatment of severe persistent asthma and chronic idiopathic or spontaneous urticaria (132, 133). It can bind to IgE with high affinity and block the binding site for FcϵRI to reduce the levels of both free IgE and peripheral eosinophils (134, 135). For patients with BP, omalizumab can reduce disease severity mainly by decreasing itching and blister counts, as well as the dose of systemic steroids (136–138). It has also shown promising therapeutic effects in refractory BP (137, 139). Ligelizumab (QGE031) is a second-generation monoclonal antibody with a higher affinity for IgE than omalizumab, which also has a significant therapeutic effect on chronic spontaneous urticaria (140). However, ligelizumab was discontinued due to insufficient efficacy in BP at phase 2 clinical trial (NCT01688882) (141). In view of the importance of IgE in the pathogenesis of BP, it will become a new target for treatment, and the research and development of more monoclonal antibodies against IgE will bring good news to patients with BP (**Table 1**).

### 3.4 Other molecule in BP

TSLP, predominantly produced by the epithelium and ILC2s when encountering stimuli, is an important initiator of type 2 inflammation and factor for itching. Multiple studies have found that the concentration of TSLP increases in skin lesions, blister fluid, and serum of patients with BP (142), and that it may be involved in the pathogenesis of BP through the direct activation of DCs (143). A study found that mice with BP180 dysfunction have increased the expression of TSLP and the latter is strongly correlated with itch severity (144). Tezepelumab, a human monoclonal antibody against TSLP, is used to treat severe and uncontrolled asthma in clinical trials and can significantly control the disease and improve quality of life (145, 146). Owing to its efficacy in asthma, tezepelumab has potential as an additive medication in BP therapy to relieve itching and improve patients’ quality of life (**Table 1**).

## 4 Discussion

BP is an autoimmune bullous disease that mainly occurs in the elderly, severely affecting their health and quality of life. Previous studies have demonstrated that BP is predominantly evoked by autoantibodies against BP180 and BP230, together with an abnormal T cell immune response, which results in the destruction of hemidesmosomes and local inflammation. Traditional therapies are mainly based on the systemic use of corticosteroids. However, long-term use of corticosteroids results in a series of side effects, such as hypertension, bone fracture, cataract, and hyperglycemia, and some patients are not sensitive to hormone therapy. Therefore, there is an urgent need for new medications with fewer side effects and superior therapeutic benefits. Type 2 inflammation is an immune response mainly mediated by ILC2s, Th2 cells, eosinophils, and inflammatory cytokines, such as IL-4, IL-5 and IL-13. As mentioned above, these immune cells and their related molecules play an essential role in the pathogenesis of BP. To date, a variety of monoclonal antibodies against the above factors have been used in the therapy of type 2 inflammation-related diseases and have achieved good clinical efficacy. From this perspective, these targeted drugs are expected to become a new and superior choice for patients with BP. However, the role and efficacy of these targeted medications in the treatment of BP is not completely clear yet; thus, more studies are needed to explore the efficacy of these targeted drugs on BP in the future to find more effective medications with fewer side effects that can benefit patients with BP.

## Author contributions

Under the supervision of XL, the manuscript was written by LZ. ZC and LW provided critical evaluation of written content and contributed to manuscript revision. All authors contributed to the article and approved the submitted version.
